# Diabetes medication associates with DNA methylation of metformin transporter genes in the human liver

**DOI:** 10.1186/s13148-017-0400-0

**Published:** 2017-09-21

**Authors:** Sonia García-Calzón, Alexander Perfilyev, Ville Männistö, Vanessa D. de Mello, Emma Nilsson, Jussi Pihlajamäki, Charlotte Ling

**Affiliations:** 1 Department of Clinical Sciences, Epigenetics and Diabetes Unit, Lund University Diabetes Centre, Jan Waldenströms gata 35, CRC 91:12, 205 02 Malmö, Sweden; 2 Institute of Clinical Medicine, Internal Medicine, University of Eastern Finland and Kuopio University Hospital, 70210 Kuopio, Finland; 3 Institute of Public Health and Clinical Nutrition, University of Eastern Finland, 80100 Joensuu, Finland; 4 Clinical Nutrition and Obesity Center, Kuopio University Hospital, 70210 Kuopio, Finland

**Keywords:** Epigenetics, Diabetes medication, Metformin, Organic cation transporters, Liver, Type 2 diabetes

## Abstract

**Background:**

Given that metformin is the most common pharmacological therapy for type 2 diabetes, understanding the function of this drug is of great importance. Hepatic metformin transporters are responsible for the pharmacologic action of metformin. However, epigenetics in genes encoding metformin transporters has not been fully elucidated. We examined the DNA methylation of these genes in the liver of subjects with type 2 diabetes and tested whether epigenetic alterations associate with diabetes medication, i.e., metformin or insulin plus metformin treatment.

**Results:**

DNA methylation in OCT1 encoded by *SLC22A1*, OCT3 encoded by *SLC22A3*, and MATE1 encoded by *SLC47A1* was assessed in the human liver. Lower average and promoter DNA methylation of *SLC22A1*, *SLC22A3*, and *SLC47A1* was found in diabetic subjects receiving just metformin, compared to those who took insulin plus metformin or no diabetes medication. Moreover, diabetic subjects receiving just metformin had a similar DNA methylation pattern in these genes compared to non-diabetic subjects. Notably, DNA methylation was also associated with gene expression, glucose levels, and body mass index, i.e., higher *SLC22A3* methylation was related to lower *SLC22A3* expression and to insulin plus metformin treatment, higher fasting glucose levels and higher body mass index. Importantly, metformin treatment did also directly decrease DNA methylation of *SLC22A1* in hepatocytes cultured in vitro*.*

**Conclusions:**

Our study supports that metformin decreases DNA methylation of metformin transporter genes in the human liver. Moreover, higher methylation levels in these genes associate with hyperglycaemia and obesity.

**Electronic supplementary material:**

The online version of this article (10.1186/s13148-017-0400-0) contains supplementary material, which is available to authorized users.

## Background

Metformin is the most common pharmacological therapy for type 2 diabetes (T2D). As metformin’s primary action is considered to be in the liver, hepatic uptake by organic cation transporters (OCT1 encoded by *SLC22A1* and OCT3 encoded by *SLC22A3*) and secretion to the bile through an efflux transporter (MATE1 encoded by *SLC47A1*) are essential for the pharmacological effect of this drug [1]. Mouse models have provided valuable insights showing the important role of metformin transporters in the liver for the pharmacological effect of metformin [2–6]. Human studies have focused on genetic variants in these transporters to elucidate their role in metformin response [7–11]. They showed that genetic variants associate with OCT1 protein and mRNA expression and OCT3 mRNA expression in the human liver [12].

Although genetics of metformin transporters have been described, epigenetic regulation of these genes is less studied. There are only two studies related to cancer assessing DNA methylation of *SLC22A1* and *SLC22A*3 in the human liver [13, 14]. Here, higher DNA methylation of *SLC22A1* was associated with decreased expression of this gene in hepatocellular carcinoma.

Given that hepatic entry of metformin is necessary for its glucose-lowering effects in patients with T2D, it would be relevant to investigate epigenetic regulation of the genes encoding metformin transporters in the human liver. Therefore, our aim was to investigate whether DNA methylation and gene expression of *SLC22A1*, *SLC22A3*, and *SLC47A1* are associated with diabetes medication in the human liver. Here, we compared diabetic patients taking metformin versus those taking insulin plus metformin or no diabetes medication as well as non-diabetic subjects. We also tested if DNA methylation in these transporters was associated with gene expression, fasting glucose levels or body mass index (BMI).

## Results

Clinical characteristics of the non-diabetic and T2D participants according to medication are shown in Additional file 1: Table S1. Diabetic subjects who were administered just metformin (*n* = 20) had lower insulin, glucose, and HOMA-IR levels compared to subjects who received insulin plus metformin (*n* = 10) or no diabetes medication (*n* = 3). Moreover, diabetic subjects who took just metformin had similar insulin and HOMA-IR levels compared to non-diabetic subjects.

**Fig. 1:**
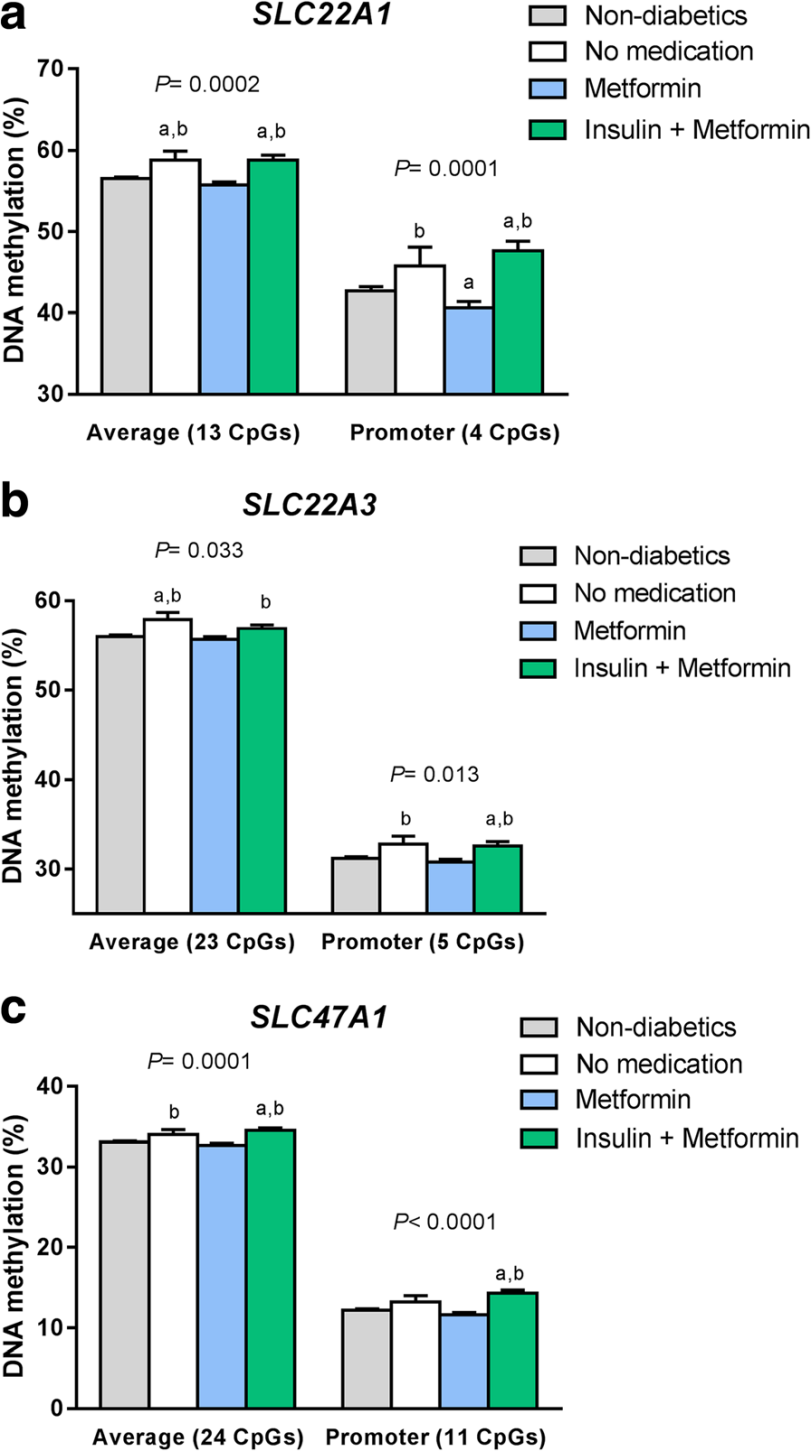
DNA methylation of metformin transporter genes in human liver of patients with type 2 diabetes (T2D) and non-diabetic subjects. **a**–**c** Average and promoter DNA methylation according to diabetes medication (20 T2D patients receiving metformin, 10 T2D patients on insulin + metformin therapy, and 3 T2D patients on no medication) and non-diabetic subjects. *P* values from the ANCOVA are shown after adjusting for age, sex, and the presence of non-alcoholic steatohepatitis (NASH). Post-hoc analyses were used to compare groups: ^a^ *P* < 0.05 compared to non-diabetic subjects, ^b^ *P* < 0.05 compared to metformin treatment. Adjusted means and standard errors are shown

We next examined if DNA methylation in the *SLC22A1*, *SLC22A3*, and *SLC47A1* genes was different in the human liver according to diabetes medication. Patients who took metformin had lower average degree of DNA methylation, especially in the promoter region, in all studied transporter genes compared to subjects who received insulin + metformin and subjects who did not receive any diabetes medication (Fig. 1a–c). Of note, in the metformin (*n* = 20) and in the insulin + metformin (*n* = 10) groups, six and two subjects respectively were also on other oral diabetes medication (Sitagliptin or Glimepiride) (Additional file 1: Table S1). However, these medications did not seem to affect methylation of *SLC22A1*, *SLC22A3*, and *SLC47A1* (Additional file 1: Table S2), and we therefore included those subjects in our analyses. Furthermore, DNA methylation in six CpG sites annotated to *SLC22A1*, one CpG site annotated to *SLC22A3* and six CpG sites annotated to *SLC47A1* were significantly different with false discovery rate (FDR) less than 5% according to diabetes medication. Notably, DNA methylation in these individual CpG sites was similar or even lower in diabetic subjects who received metformin compared to non-diabetic individuals (Table 1).

**Table 1:** Individual CpG sites in metformin transporter genes that exhibit differential DNA methylation in human liver according to diabetes medication and non-diabetic subjects (*n* = 93)

Probe ID	Chr	Position	Gene region	Non-diabetics (*n* = 60)	No medication (*n* = 3)	Metformin (*n* = 20)	Insulin + Metformin (*n* = 10)	*P-*ANCOVA	*q* value
*SLC22A1*									
cg13434757	6	160541976	TSS1500	52.0 ± 0.8	56.0 ± 3.5	48.8 ± 1.3^a^	56.2 ± 1.9^a, b^	0.010	0.022
cg05314142	6	160542711	TSS200	41.7 ± 0.7	43.2 ± 3.3	38.9 ± 1.2	46.3 ± 1.7^a, b^	0.009	0.022
cg24864413	6	160542732	TSS200	40.3 ± 0.5	44.5 ± 2.3^b^	39.5 ± 0.9	47.3 ± 1.2^a, b^	<0.0001	**<** 0.0001
cg22416916	6	160542770	TSS200	36.6 ± 0.5	39.5 ± 2.1	35.2 ± 0.8	40.7 ± 1.1^a, b^	0.002	0.013
cg13466809	6	160542944	1stExon;5’UTR	35.5 ± 0.4	36.5 ± 2.0	34.3 ± 0.7	39.0 ± 1.1^a, b^	0.007	0.022
cg27292431	6	160543261	1stExon	43.2 ± 0.6	46.9 ± 2.9	42.1 ± 1.2	48.4 ± 1.5^a, b^	0.008	0.022
Average DNA methylation (13 CpG sites)				56.5 ± 0.2	58.8 ± 1.1^a, b^	55.7 ± 0.4	58.8 ± 0.6^a, b^	0.0002	
DNA methylation promoter region : TSS1500 and TSS200 (4 CpG sites)				42.7 ± 0.5	45.8 ± 2.3^b^	40.6 ± 0.8^a^	47.6 ± 1.2^a, b^	0.0001	
*SLC22A3*									
cg22117918	6	160769114	TSS1500	8.3 ± 0.2	9.1 ± 0.9	7.9 ± 0.3	9.7 ± 0.5^a, b^	0.033	0.138
cg25313204	6	160768801	TSS1500	42.9 ± 0.6	48.2 ± 2.8	42.9 ± 1.0	47.8 ± 1.5^a, b^	0.014	0.107
cg06295784	6	160771074	Body	40.7 ± 0.5	44.3 ± 2.5	39.6 ± 10.9	44.8 ± 1.3^a, b^	0.012	0.107
cg13280882	6	160771574	Body	77.3 ± 1.4	91.9 ± 6.4^a, b^	77.3 ± 2.4	85.9 ± 3.4^a, b^	0.033	0.138
cg11696576	6	160815734	Body	45.1 ± 0.6	52.0 ± 2.7^a, b^	42.3 ± 1.0^a^	49.2 ± 1.4^a, b^	0.0002	0.005
cg04794858	6	16086035	Body	91.3 ± 0.3	89.6 ± 1.3	90.9 ± 0.5	89.0 ± 0.7^a, b^	0.036	0.138
Average DNA methylation (23 CpG sites)				56.0 ± 0.2	57.9 ± 0.8^a, b^	55.7 ± 0.3	56.9 ± 0.4^b^	0.033	
DNA methylation promoter region: TSS1500 and TSS200 (5 CpG sites)				31.2 ± 0.2	32.8 ± 0.9^b^	30.8 ± 0.3	32.6 ± 0.5^a, b^	0.013	
*SLC47A1*									
cg01530032	17	19435805	TSS1500	31.7 ± 0.7	38.5 ± 3.0^a, b^	30.6 ± 1.1	39.7 ± 1.6^a, b^	0.0001	0.001
cg25387636	17	19436896	TSS1500	7.4 ± 0.2	8.0 ± 1.1	6.3 ± 0.4^a^	8.5 ± 0.6^b^	0.019	0.057
cg15971010	17	19436900	TSS1500	10.1 ± 0.3	11.2 ± 1.5	8.6 ± 0.5^a^	12.0 ± 0.8^a, b^	0.007	0.028
cg15014549	17	19437003	TSS200	3.7 ± 0.7	3.7 ± 0.3	3.8 ± 0.1	4.4 ± 0.2^a, b^	0.007	0.028
cg07829432	17	19437013	TSS200	3.8 ± 0.1	4.7 ± 0.4	3.8 ± 0.2	5.2 ± 0.2^a, b^	<0.0001	**<** 0.0001
cg20930201	17	19437691	Body	7.7 ± 0.2	9.2 ± 0.9^b^	6.7 ± 0.3^a^	9.1 ± 0.5^a, b^	0.0009	0.007
cg26959235	17	19437889	Body	12.7 ± 0.2	11.9 ± 1.1	12.7 ± 0.4	10.8 ± 0.6^a, b^	0.033	0.086
cg24151087	17	19450271	Body	9.9 ± 0.2	10.5 ± 1.2	9.8 ± 0.4	12.1 ± 0.6^a, b^	0.018	0.057
cg16887170	17	19451180	Body	84.0 ± 0.4	83.3 ± 1.8	81.7 ± 0.7^a^	83.0 ± 0.9	0.036	0.086
cg12550399	17	19482275	3′ UTR	64.2 ± 0.8	67.0 ± 3.6	62.8 ± 1.3	71.9 ± 1.9^a, b^	0.002	0.011
Average DNA methylation (24 CpG sites)				33.1 ± 0.1	34.0 ± 0.6^b^	32.7 ± 0.2	34.5 ± 0.3^a, b^	0.0001	
DNA methylation promoter region: TSS1500 and TSS200 (11 CpG sites)				12.2 ± 0.2	13.2 ± 0.8	11.6 ± 0.3	14.3 ± 0.4^a, b^	<0.0001	

Data are shown as mean ± SEM. All data is adjusted for age, sex, and NASH. Post-hoc analysis is used for comparisons between groups after ANCOVA. *q* values are based on false discovery rate (FDR) tests after ANCOVA

^a^ *P* < 0.05 compared to non-diabetic subjects

^b^ *P* < 0.05 compared to metformin treatment

**Fig. 2:**
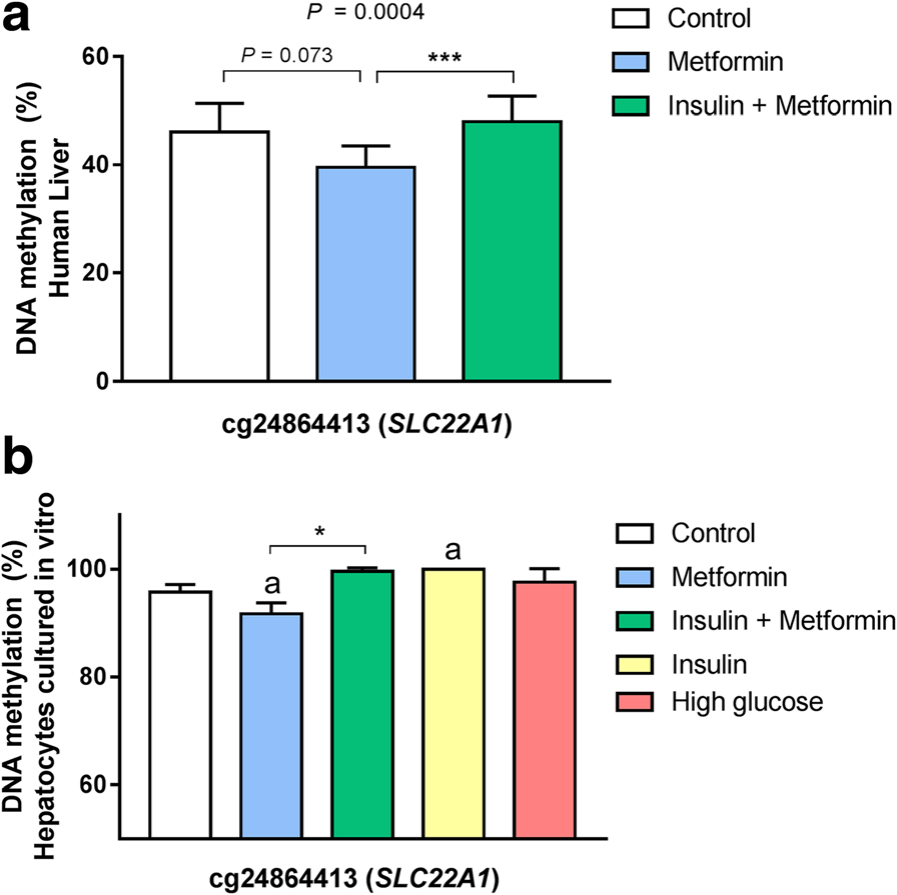
DNA methylation in the promoter region of *SLC22A1* (cg24864413). **a** DNA methylation of *SLC22A1* in human liver was lower in type 2 diabetes subjects receiving just metformin (*n* = 20), compared to those who took insulin plus metformin (*n* = 10) or no diabetes medication (*n* = 3). *P* value from the ANCOVA is shown after adjusting for age, sex, and the presence of non-alcoholic steatohepatitis (NASH). Post-hoc analyses were used to compare groups: ***P < 0.0001. Adjusted means and standard errors are shown. **b** DNA methylation of *SLC22A1* in hepatocytes cultured in vitro was lower after 8 h of metformin treatment (0.5 mM) compared to insulin plus metformin treatment and to control Huh-7 cells, whereas insulin treatment (100 nM) increased DNA methylation of this CpG site (*n* = 4). *P < 0.05, ^a^ *P* < 0.05 compared to control, all analyzed by a paired *t* test. Means and standard deviations are shown

We next tested whether metformin and/or insulin treatment might affect DNA methylation in metformin transporter genes directly in cell cultures. Here, methylation of three CpG sites (cg24864413, cg11696576, cg01530032) was analyzed by pyrosequencing. These sites were selected as they were the most significant sites for each of the three studied genes (Table 1) and had relatively large differences in methylation by diabetes medication in the human liver. In agreement with the liver data (Fig. 2a), cg24864413 annotated to *SLC22A1* had lower methylation in cells treated with metformin compared to insulin + metformin (*P* = 0.016) and to untreated cells (*P* = 0.025) (Fig. 2b). Moreover, insulin treatment alone also increased methylation in this CpG site in the cells (*P* = 0.035). No differences in DNA methylation were observed for the other two CpG sites (Additional file 1: Figure S1).

We further related DNA methylation to gene expression of the studied metformin transporters in the human liver of 42 subjects. Pearson correlations showed that liver DNA methylation in some individual CpG sites (one CpG at the *SLC22A1* locus, three CpGs at the *SLC22A3* locus, and one CpG at the *SLC47A1* locus) was associated with expression of its corresponding gene (*P* < 0.05) (Table 2). These associations remained after adjusting for age, sex, and the presence of non-alcoholic steatohepatitis (NASH). However, while *SLC22A1* and *SLC47A1* had higher expression than *SLC22A3*, no expression differences were observed for any of the three metformin transporters according to diabetes medication (Additional file 1: Figure S2).

**Table 2:** Correlations between DNA methylation and gene expression of metformin transporter genes in human liver with *P* value < 0.05 in subjects from the Kuopio Obesity Surgery Study (*n* = 42)

Probe ID	Chr.	Position	Gene region	*r* (*P* value)	*q* value	*B* (*P* value)^a^
*SLC22A1*						
cg05307864	6	160559487	Body	0.408 (0.007)	0.091	0.64 (0.011)
*SLC22A3*						
cg02042585	6	160783785	Body	0.409 (0.007)	0.161	4.40 (0.010)
cg06295784	6	160771074	Body	− 0.368 (0.016)	0.184	− 3.47 (0.026)
cg17364114	6	160769359	TSS200	0.320 (0.038)	0.291	2.76 (0.024)
*SLC47A1*						
cg12799818	17	19450343	Body	0.338 (0.029)	0.696	0.89 (0.036)

*q* values are based on false discovery rate (FDR) tests

*r* correlation coefficient, *B* regression coefficient

^a^Adjusted for age, sex, and NASH

We also studied whether liver DNA methylation in the metformin transporter genes was related to glucose levels or BMI in the 95 subjects from the Kuopio Obesity Surgery Study (Table 3). Glucose levels and BMI were positively correlated with the degree of average methylation of *SLC22A1* and *SLC47A1* and methylation in the promoter region of *SLC22A1*, *SLC22A3*, and *SLC47A1.* Higher DNA methylation of cg24864413 (*SLC22A1*), cg06295784 (*SLC22A3*), cg07883823 (*SLC22A3*), cg01530032 (*SLC47A1*), cg07829432 (*SLC47A1*) and cg12550399 (*SLC47A1*) was associated with higher glucose levels (*q* value < 0.05). Moreover, DNA methylation in cg13466809 (*SLC22A1*), cg06295784 (*SLC22A3*), and cg25313204 (*SLC22A3*) showed positive correlations with BMI (*q* value < 0.001).

**Table 3:** Correlations between DNA methylation of metformin transporter genes in human liver and metabolic phenotypes including fasting glucose and BMI with *P* values < 0.05 in subjects from the Kuopio Obesity Surgery Study (*n* = 95)

Probe ID	Chr.	Position	Gene region	*B* (*P* value)	*q* value
GLUCOSE (mmol/L)^a^					
*SLC22A1*					
cg24864413	6	160542732	TSS200	0.73 (0.001)	0.013
cg22416916	6	160542770	TSS200	0.52 (0.011)	0.065
cg13466809	6	160542944	1stExon;5’UTR	0.39 (0.027)	0.070
cg27292431	6	160543261	1stExon	0.61 (0.020)	0.065
cg07558837	6	160555312	Body	0.22 (0.015)	0.065
Average DNA methylation (13 CpG sites)				0.33 (0.002)	
DNA methylation promoter region: TSS1500 and TSS200 (4 CpG sites)				0.60 (0.007)	
*SLC22A3*					
cg06295784	6	160771074	Body	0.62 (0.004)	0.046
cg07883823	6	160769116	TSS1500	0.30 (0.002)	0.046
cg11696576	6	160815734	Body	0.57 (0.025)	0.143
cg22117918	6	160769114	TSS1500	0.16 (0.043)	0.198
cg25313204	6	160768801	TSS1500	0.65 (0.008)	0.061
Average DNA methylation (23 CpG sites)				0.14 (0.056)	
DNA methylation promoter region: TSS1500 and TSS200 (5 CpG sites)				0.21 (0.008)	
*SLC47A1*					
cg01530032	17	19435805	TSS1500	0.91 (0.002)	0.016
cg07829432	17	19437013	TSS200	0.13 (0.001)	0.012
cg08895056	17	19438253	Body	0.49 (0.031)	0.136
cg11784214	17	19479935	Body	0.19 (0.047)	0.161
cg12550399	17	19482275	3′ UTR	1.14 (0.001)	0.012
cg15014549	17	19437003	TSS200	0.06 (0.034)	0.136
cg24151087	17	19450271	Body	0.24 (0.020)	0.120
Average DNA methylation (24 CpG sites)				0.16 (0.005)	
DNA methylation promoter region: TSS1500 and TSS200 (11 CpG sites)				0.20 (0.012)	
BMI (kg/m^2^)^b^					
*SLC22A1*					
cg13434757	6	160541976	TSS1500	0.22 (0.045)	0.230
cg13466809	6	160542944	1stExon;5′UTR	0.23 (<0.0001)	*<0.0001*
Average DNA methylation (13 CpG sites)				0.09 (0.013)	
DNA methylation promoter region: TSS1500 and TSS200 (4 CpG sites)				0.16 (0.038)	
*SLC22A3*					
cg06295784	6	160771074	Body	0.28 (<0.0001)	*<0.0001*
cg09226986	6	160852328	Body	− 0.12 (0.020)	0.153
cg25313204	6	160768801	TSS1500	0.31 (<0.0001)	*<0.0001*
Average DNA methylation (23 CpG sites)				0.03 (0.261)	
DNA methylation promoter region: TSS1500 and TSS200 (5 CpG sites)				0.06 (0.030)	
*SLC47A1*					
cg01530032	17	19435805	TSS1500	0.22 (0.029)	0.174
cg10718608	17	19438221	Body	0.20 (0.003)	0.072
cg12133118	17	19436770	TSS1500	0.17 (0.006)	0.072
cg12550399	17	19482275	3′ UTR	0.26 (0.024)	0.174
Average DNA methylation (24 CpG sites)				0.06 (0.001)	
DNA methylation promoter region: TSS1500 and TSS200 (11 CpG sites)				0.06 (0.025)	

*q* values are based on false discovery rate (FDR) tests

*B* regression coefficient

^a^Adjusted for age and sex

^b^Adjusted for age, sex, and NASH

**Fig. 3:**
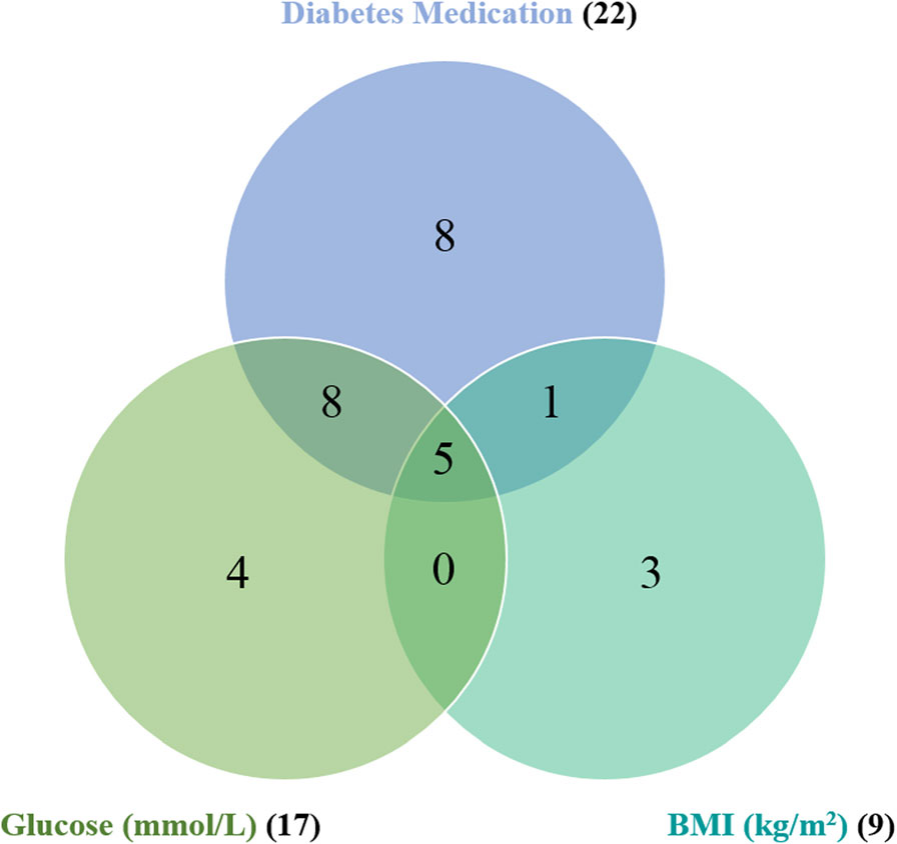
Venn diagram showing associations between DNA methylation of the three metformin transporters genes and diabetes medication, glucose levels, and body mass index (BMI). Overall, DNA methylation of 29 CpG sites was associated with either diabetes medication, glucose levels or BMI with a *P* value < 0.05. An overlap with differential DNA methylation in five CpG sites was observed for the three factors

A Venn diagram was finally made to show the associations between DNA methylation of metformin transporters and diabetes medication, glucose levels and BMI (Fig. 3). An overlap including DNA methylation in five CpG sites was found for the three factors, i.e., cg13466809 (*SLC22A1*)*,* cg06295784 (*SLC22A3*), cg25313204 (*SLC22A3*), cg01530032 (*SLC47A1*), and cg12550399 (*SLC47A1*) displayed lower DNA methylation levels in the metformin group and were positively associated with glucose and BMI.

## Discussion

In this study, we describe DNA methylation and gene expression of *SLC22A1*, *SLC22A3*, and *SLC47A1*, which respectively encode the three metformin transporters OCT1, OCT3, and MATE1, in the human liver. In agreement with a previous study where OCT1 was found to be the most expressed drug transporter in the liver [15], we found higher mRNA expression of *SLC22A1* than *SLC22A3* (Additional file 1: Figure S2A). *SLC47A1* was also highly expressed in the liver, as previously reported [16].

Metformin transporter genes have been studied in vivo in rodents to explain metformin pharmacodynamics. The distribution of metformin to the liver in Oct1 −/− mice was reduced 30-fold compared with wild-type mice [6], and the glucose-lowering effects of metformin were totally abolished in the knock out mice [3]. Liver accumulation of metformin was also reduced in Oct3 −/− mice [2]. These results support that OCT1/*SLC22A1* and OCT3/*SLC22A3* are responsible for hepatic uptake of metformin. In contrast, the lack of *SLC47A1* expression led to 69-fold higher metformin concentrations in the liver [4, 5], confirming the role that MATE1/*SLC47A1* plays in hepatic excretion of metformin. Indeed, liver expression of these metformin transporter genes is crucial for the antidiabetic effect of metformin.

Recent studies from our group and others have shown that epigenetics play an important role in the pathogenesis of T2D in different tissues, including the human liver [17–21]. But, it is uncertain whether epigenetics is associated with diabetes medication, such as metformin or insulin. This is the first study reporting differential DNA methylation in metformin transporter genes in the human liver according to diabetes medication. Subjects who just received metformin presented lower methylation levels, mainly in the promoter region, in all three transporter genes compared to those participants who were taking insulin plus metformin or no diabetes medication. To dissect if diabetes medication has a direct impact on methylation, we exposed liver cells to metformin and insulin in vitro. Interestingly, methylation of *SLC22A1* was lower in cells exposed to metformin and higher after insulin treatment, which is in line with the data seen in the human liver. The fact that methylation of some studied CpG sites was not affected by the exposure in vitro does not exclude that longer metformin or insulin treatments could have effects. T2D patients are given a long-term therapy, whereas the cells were treated for 8 h mimicking an acute therapy. Overall, our in vivo and in vitro data support that metformin therapy is associated with lower DNA methylation of metformin transporter genes in the liver suggesting that epigenetics could be a potential mechanism for metformin action in the human liver. Accordingly, a recent study has shown that metformin alters DNA methylation in endometrial cancer cells [22]. The demethylation process in metformin transporters induced by metformin could have occurred passively or actively by ten-eleven translocation (TET) enzymes [23], since these enzymes play an important role in the development and function of the human liver [24]. Moreover, AMPK pathway, activated by metformin, elevates α-ketoglutarate metabolite which is required by TET catalytic reaction for the DNA demethylation process [25]. Nevertheless, more studies are needed to dissect this mechanism. In addition, diabetics who were only on metformin therapy had a similar or lower degree of methylation in these metformin transporter genes compared to non-diabetic people, suggesting a possible normalization of the DNA methylation status in these people. However, it is also possible that insulin increases methylation in metformin transporter genes since both the control and the insulin + metformin groups presented higher serum insulin levels. Additionally, insulin treatment increased methylation in vitro. It should be noted that we used a lower metformin concentration (0.5 mM) than other in vitro studies (5–10 mM) [26] to be closer to the physiological level seen in humans (~ 50 uM in plasma from T2D subjects) [26].

Moreover, we observed that higher DNA methylation in these transporters was associated with higher glucose levels and BMI. Higher methylation and lower expression of *SLC22A1* and *SLC22A3* was previously observed in hepatocellular carcinoma and prostate tumor compared with matched normal samples [13, 14] supporting that higher methylation in metformin transporter genes could be associated with disease. However, there are no further studies assessing DNA methylation in metformin transporters to compare with our results.

The clinical response to metformin shows considerable inter-individual variation. Human studies found genetic variants in *SLC22A1*, *SLC22A3*, and *SLC47A1* that are associated with an impaired metformin transport and a poor glycaemic response to metformin [7–11]. One study also showed that genetic variants influence OCT1 and OCT3 expression and function in the human liver [12]. Epigenetic factors may also regulate gene expression [27]. We observed that methylation of some sites near to these genes correlated with expression, supporting that methylation in metformin transporter genes is related with expression in the human liver. For example, higher methylation in a CpG site located in *SLC22A3* was associated with lower expression in the human liver and also with insulin plus metformin therapy, higher glucose levels, and BMI. Higher DNA methylation of *SLC22A3* could be a potential mechanism to decrease expression of this gene, leading to reduced antidiabetic effects of metformin resulting in hyperglycaemia. DNA methylation was initially thought to be a silencing mark. However, merging data show that the role of DNA methylation is much more complex than initially thought, and it is dependent on genomic location and can be dependent on several other factors [27]. DNA methylation in the gene body has been associated with increased gene expression potentially by increased elongation, while methylation in the promoter region often associate with decreased transcriptional activity [28]. DNA methylation may also regulate expression of non-coding RNAs, alternative splicing events, and the overall genomic stability [27]. Recent studies also point out that CpG sites are positively or negatively associated to gene expression depending on other epigenetic marks such as histone marks and chromatin accessibility [29]. These are likely reasons for why we found positive and negative correlations between DNA methylation of individual CpG sites in metformin transporters and gene expression.

The low number of patients with no diabetes medication could be a limitation in this study. However, the fact that statistical differences were found suggests that potential type II errors were overcome. Another limitation of this study is the lack of genetics data that may provide evidence for interactions between genetic and epigenetic events of metformin transporter genes in the liver. Nevertheless, our main aim was to assess DNA methylation of metformin transporter genes which has been scarcely studied.

## Conclusions

We show for the first time that DNA methylation in metformin transporter genes in the human liver is different according to diabetes medication and associates with gene expression. Lower methylation in *SLC22A1*, *SLC22A3*, and *SLC47A1* in the liver was associated with metformin therapy, lower glucose levels, and lower BMI. This study shows a novel mechanism of metformin which regulates the epigenetic pattern of the key metformin transporters in the human liver.

## Methods

### Study participants and clinical characteristics

This study includes 95 obese participants (64% women, age 49.5 ± 7.7 years, 35 with T2D and 26 subjects with NASH who were recruited from the Kuopio Obesity Surgery Study (Additional file 1: Table S3). The participants underwent Roux-en-Y gastric bypass surgery and liver biopsies were collected during the operation. Informed consent was obtained and the study protocol was approved by the Ethics Committee of Northern Savo Hospital District [30, 31]. All experiments were performed in accordance with the relevant guidelines and regulations.

Fasting blood samples were drawn on the morning of the surgery, and plasma glucose and serum insulin were analyzed as published elsewhere [32]. Data regarding body weight, height, and presence of diabetes, simple steatosis or NASH were also collected [32]. Three diabetic patients who did not receive any diabetes medication and 30 T2D patients who were taking metformin or insulin plus metformin were considered for the analyses (Additional file 1: Table S1). Among these, eight subjects were also on other oral diabetes medication (Sitagliptin or Glimepiride). In addition, 60 non-diabetic subjects were included in some analyses.

### DNA methylation analysis

DNA methylation was assessed in all CpG sites annotated to three metformin transporter genes on the Infinium HumanMethylation450 BeadChip from Illumina, including 13 sites annotated to *SLC22A1*, 23 sites annotated to *SLC22A3* and 24 sites annotated to *SLC47A1*. The genome-wide DNA methylation data from the human liver has previously been published and included 95 participants whose characteristics are presented in Additional file 1: Table S3 [20].

### Gene expression analysis

mRNA expression of *SLC22A1*, *SLC22A3*, and *SLC47A1* was analyzed in the human liver using HumanHT-12 Expression BeadChip (Illumina). mRNA expression was assessed in the liver from a subset of subjects (42 participants) due to the limited size of human liver biopsies, available amounts of liver RNA, and resources [20]. Among these 42 participants, 19 were diabetics of whom 13 were receiving metformin, 5 were on insulin plus metformin therapy, and 1 was not on any diabetes medication. The characteristics of these 42 participants have previously been described [20].

### Cell culture experiments

Huh-7 human hepatocellular carcinoma cells were cultured with DMEM, 1.0 g/L glucose plus 10% FBS and 1% penicillin/streptomycin. Cells were treated for 8 h with either 0.5 mM metformin (Sigma-Aldrich), 100 nM insulin, 0.5 mM metformin plus 100 nM insulin, or 24 mM glucose. The treatment time was selected since it has previously been shown that metformin treatment for 8 h in vitro has an effect on gene expression and activates AMPK phosphorylation [33, 34].

Genomic DNA was extracted from cells using DNeasy blood and tissue kit (Qiagen), and DNA methylation was analyzed using PyroSequencing as previously reported [18]. PyroMark Assay Design Software 2.0 (Qiagen) was used to design PyroSequencing primers (Additional file 1: Table S4).

### Statistical analyses

Participant’s clinical characteristics were compared according to diabetes medication using the one-way ANOVA test. ANCOVA test was used to assess DNA methylation or gene expression levels according to diabetes medication, followed by post-hoc tests to compare groups. Paired *t* test was used for in vitro experiments. DNA methylation was correlated to expression levels using Pearson correlations and linear regression models. To assess associations between DNA methylation and glucose levels or BMI, a linear regression model was used. ANCOVA and linear models were adjusted for age, sex, and NASH except for glucose associations where just age and sex were included because there was a high correlation between NASH and glucose levels *(P* = 0.0029). To separate the effect of medication from NASH on DNA methylation, we analyzed the residuals without including diabetes medication in the model (age, sex, and NASH were included). The residuals did not depend on NASH (*P* value > 0.05) indicating that NASH and medication likely are independent variables and that the model has been well corrected for NASH. FDR was used to correct for multiple testing. Statistical analyses were performed using STATA v12.0 (StataCorp).

## Additional file

Additional file 1: Table S1. Clinical characteristics of the 33 type 2 diabetic patients according to drug treatment and of 60 non-diabetic subjects. **Table S2.** Average and promoter DNA methylation according to diabetes medication, first comparing other oral medication such as Sitagliptin or Glimepiride + metformin to only metformin therapy, and second excluding all subjects that were receiving Sitagliptin or Glimepiride. **Table S3.** Clinical characteristics of subjects from the Kuopio Obesity Surgery Study. **Table S4.** DNA sequences of the primers used for pyrosequencing. **Figure S1.** DNA methylation of *SLC22A3* and *SLC47A1* in hepatocytes cultured in vitro after 8 h of metformin (0.5 mM), insulin plus metformin, insulin (100 nM) or glucose treatment in Huh-7 cells (*n* = 4). Means and standard deviations are shown, and paired *t* test was used for the analysis. **Figure S2.** Gene expression levels of the three metformin transporter genes in the human liver (A) (*n* = 42); gene expression of transporter genes according to diabetes medication after adjusting for age, sex and non-alcoholic steatohepatitis (NASH) (B). ns: no significant. (DOCX 213 kb)

## Abbreviations

BMI: Body mass index
NASH: Non-alcoholic steatohepatitis
T2D: Type 2 diabetes
