# Supplementary material for: Diabetes medication associates with DNA methylation of metformin transporter genes in the human liver
Source: Clin Epigenetics. 2017 Sep 21;9:102. doi: 10.1186/s13148-017-0400-0 (PMC5609005; doi:10.1186/s13148-017-0400-0)
Supplement: Additional file 1: Table S1. — Clinical characteristics of the 33 type 2 diabetic patients according to drug treatment and of 60 non-diabetic subjects. Table S2. Average and promoter DNA methylation according to diabetes medication, first comparing other oral medication such as Sitagliptin or Glimepiride + metformin to only metformin therapy, and second excluding all subjects that were receiving Sitagliptin or Glimepiride. Table S3. Clinical characteristics of subjects from the Kuopio Obesity Surgery Study. Table S4. DNA sequences of the primers used for pyrosequencing. Figure S1. DNA methylation of SLC22A3 and SLC47A1 in hepatocytes cultured in vitro after 8 h of metformin (0.5 mM), insulin plus metformin, insulin (100 nM) or glucose treatment in Huh-7 cells (n = 4). Means and standard deviations are shown, and paired t test was used for the analysis. Figure S2. Gene expression levels of the three metformin transporter genes in the human liver (A) (n = 42); gene expression of transporter genes according to diabetes medication after adjusting for age, sex and non-alcoholic steatohepatitis (NASH) (B). ns: no significant. (DOCX 213 kb) [file 13148_2017_400_MOESM1_ESM.docx]

|  | **Non-diabetics (n= 60)** | **No medication (n=3)** | **Metformin**  **(n=20)** | **Insulin + Metformin**  **(n=10)** | **P-ANOVA** |
| --- | --- | --- | --- | --- | --- |
| Age (years) | 48.9 ± 7.9 | 55.2 ± 1.6 | 50.9 ± 7.3 | 50.4 ± 6.2 | 0.411 |
| Females/Males | 17 /43 | 1 / 2 | 9 / 11 | 6 / 4 | 0.118 |
| BMI (kg/m^2^) | 43.6 ± 5.5 | 37.2 ± 3.5 | 42.0 ± 5.8 | 42.8 ± 5.9 | 0.226 |
| fP-Glucose levels (mmol/L) | 5.8 ± 0.8 | 9.3 ± 4.6 ^a,b^ | 6.7 ± 1.8 ^a^ | 8.1 ± 2.8  ^a,b^ | <0.0001 |
| fS-Insulin (mU/L) | 16.2 ± 8.9 | 95.4 ± 139.3 ^a,b^ | 16.6 ± 110.9 | 115.3 ± 171.2 ^a,b^ | <0.0001 |
| HOMA-IR | 4.3 ± 2.7 | 58.3 ± 93.4 ^a,b^ | 4.6 ± 2.5 | 52.3 ± 86.5 ^a,b^ | <0.0001 |

**Table S1.** Clinical characteristics of the 33 type 2 diabetic patients according to drug treatment and of 60 non-diabetic subjects.

Abbreviations: fP, fasting plasma; fS, fasting serum

Data are shown as mean ± SD.

^*^In the metformin and in the Insulin + metformin groups, 6 and 2 subjects respectively were also on other oral diabetes medication (Sitagliptin or Glimepiride).

^a^ *P*<0.05 compared to non-diabetic subjects, ^b^ *P*<0.05 compared to metformin treatment.

|  | **Metformin**  **(n=14)** | **Sitagliptin or Glimepiride +**  **Metformin**  **(n=6)** | ***P*-value^*^** | **Metformin**  **(n=14)** | **Insulin**  **+**  **Metformin**  **(n=8)** | ***P*-value^†^** |
| --- | --- | --- | --- | --- | --- | --- |
| ***SLC22A1*** |  |  |  |  |  |  |
| Average | 55.5 ± 0.5 | 56.3 ± 0.8 | 0.413 | 55.7 ± 0.5 | 58.9 ± 0.7 | 0.003 |
| Promoter | 40.2 ± 0.9 | 41.9 ± 1.5 | 0.370 | 40.5 ± 1.2 | 48.3 ± 1.6 | 0.002 |
| ***SLC22A3*** |  |  |  |  |  |  |
| Average | 55.9 ± 0.4 | 55.8 ± 0.7 | 0.885 | 55.9 ± 0.4 | 57.1 ± 0.5 | 0.108 |
| Promoter | 30.9 ± 0.4 | 30.7 ± 0.6 | 0.748 | 31.2 ± 0.4 | 32.3 ± 0.5 | 0.095 |
| ***SLC47A1*** |  |  |  |  |  |  |
| Average | 32.7 ± 0.2 | 32.9 ± 0.3 | 0.724 | 32.7 ± 0.3 | 34.6 ± 0.4 | 0.001 |
| Promoter | 11.6 ± 0.3 | 11.7 ± 0.5 | 0.846 | 11.5 ± 0.3 | 14.2 ± 0.4 | 0.0002 |

**Table S2.** Average and promoter DNA methylation according to diabetes medication, first comparing other oral medication such as Sitagliptin or Glimepiride + metformin to only metformin therapy, and second excluding all subjects that were receiving Sitagliptin or Glimepiride.

Data shows adjusted means (± SEM) including the following covariates: age, sex and the presence of non-alcoholic steatohepatitis (NASH).

**^*^** Metformin vs. metformin + Sitagliptin or Glimepiride, excluding subjects that were receiving insulin.

**^†^** Metformin vs. metformin + insulin, excluding subjects that were receiving other oral medication such as Sitagliptin or Glimepiride.

|  | **All participants (n=95)** |
| --- | --- |
| Age (years) | 49.5 ± 7.7 |
| Females/Males | 61 / 34 |
| BMI (kg/m^2^) | 43.0 ± 5.7 |
| fP-Glucose levels (mmol/L) | 6.5 ± 2.0 |
| fS-Insulin (mU/L) | 32.7 ± 74.5 |
| Diabetes (Yes^1^ / No) | 35 / 60 |
| NASH (Normal liver / Simple Steatosis / NASH) | 35 / 34 / 26 |
| Medication ( Metformin / Insulin + Metformin)^2^ | 20 /10 |

**Table S3.** Clinical characteristics of subjects from the Kuopio Obesity Surgery Study.

Abbreviations: fP, fasting plasma; fS, fasting serum; NASH, Non-alcoholic steatohepatitits

Data are shown as mean ± SD.

^1^ Among the diabetics: 30 were taking metformin or insulin plus metformin, 3 did not receive any diabetes medication and 2 were not on metformin therapy but on other oral antidiabetic medication.

^2^ In the metformin and in the Insulin + metformin groups, 6 and 2 subjects respectively were also on other oral diabetic medication (Sitagliptin or Glimepiride).

**Table S4**. DNA sequences of the primers used for pyrosequencing

| **Gene** | **Probe ID** | **Forward primer** | **Reverse primer (5' biotinylated)** | **Sequencing primer** |
| --- | --- | --- | --- | --- |
| *SLC22A1* | cg24864413 | 5'-TGAGGGAGTTATTAGGAGGTTTGTA-3' | 5'-CTAAAAAACAACCAAATAAAAATAATCTTT-3' | 5'-TTTGTGGTTGAATTTTAATTTTT-3' |
| *SLC22A3* | cg11696576 | 5'-GGTTAGGTATTTTAATTAGAGAGGGATTTA-3' | 5'-CCAACAAAACAAAACACTACAAAAACTAT-3' | 5'-ATTTTAATTAGAGAGGGATTTAATA-3' |
| *SLC47A1* | cg01530032 | 5'-GAGGAGGGGAGGGTTTTATTTTT-3' | 5'-ACAACCTTTCTCTTCCTATACAAA-3' | 5'-AGGGTTTTATTTTTTGGT-3' |

**Figure S1.** DNA methylation of *SLC22A3* and *SLC47A1* in hepatocytes cultured in vitro after 8 hours of metformin (0.5 mM), insulin plus metformin, insulin (100 nM) or glucose treatment in Huh-7 cells (n=4). Means and standard deviations are shown and paired *t*-test was used for the analysis.


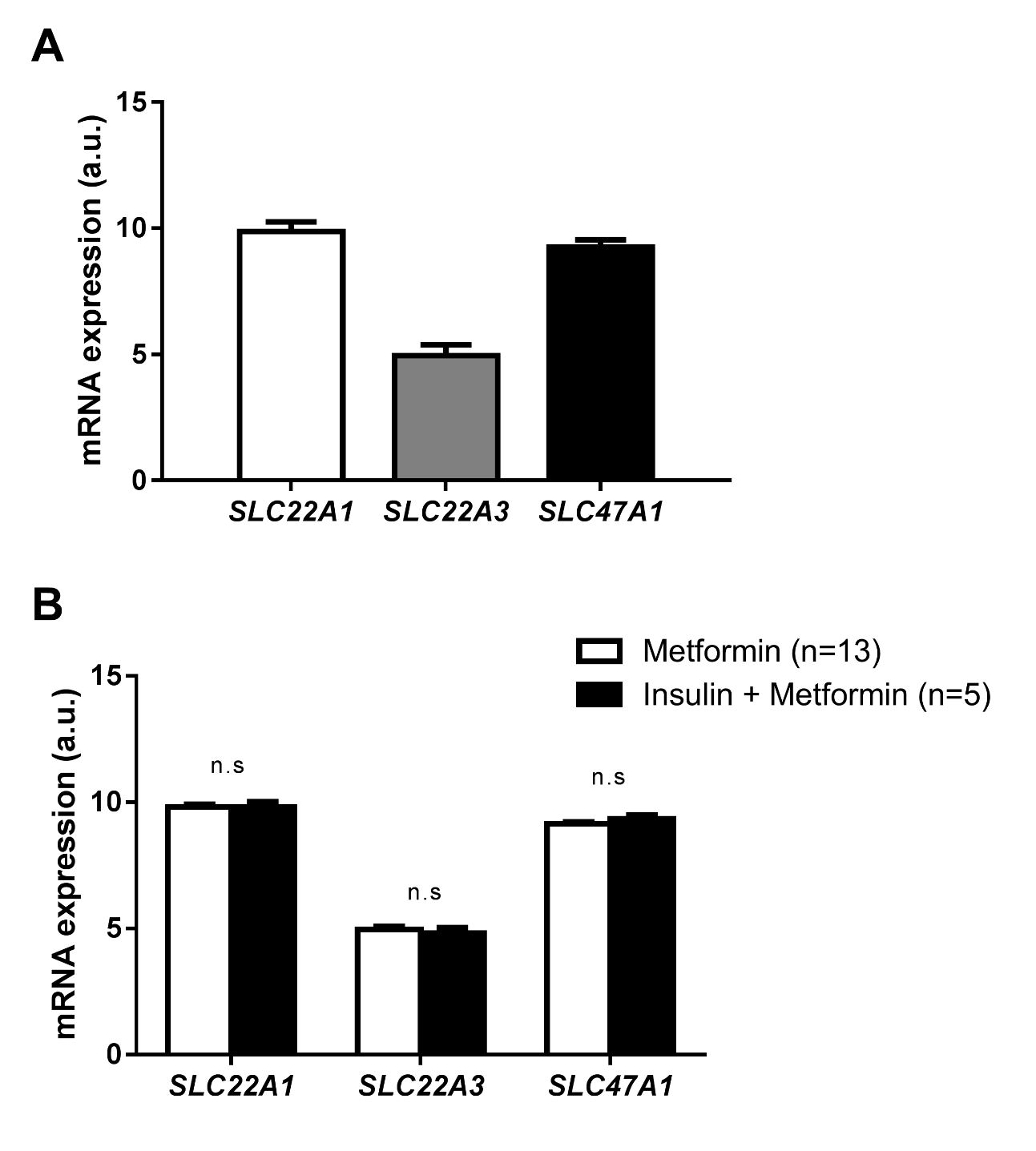


**Figure S2.** Gene expression levels of the three metformin transporter genes in human liver (**A**) (n=42); gene expression of transporter genes according to diabetes medication after adjusting for age, sex and non-alcoholic steatohepatitis (NASH) (**B**). ns: no significant.
